# HyperPCM: Robust Task-Conditioned Modeling of Drug–Target Interactions

**DOI:** 10.1021/acs.jcim.3c01417

**Published:** 2024-01-08

**Authors:** Emma Svensson, Pieter-Jan Hoedt, Sepp Hochreiter, Günter Klambauer

**Affiliations:** † ELLIS Unit Linz & Institute for Machine Learning, Johannes Kepler University, Linz 4040, Austria; ‡ Molecular AI, Discovery Sciences, R&D, AstraZeneca, Gothenburg, 431 83, Sweden; ¶ Institute of Advanced Research in Artificial Intelligence (IARAI), Vienna 1030, Austria

## Abstract

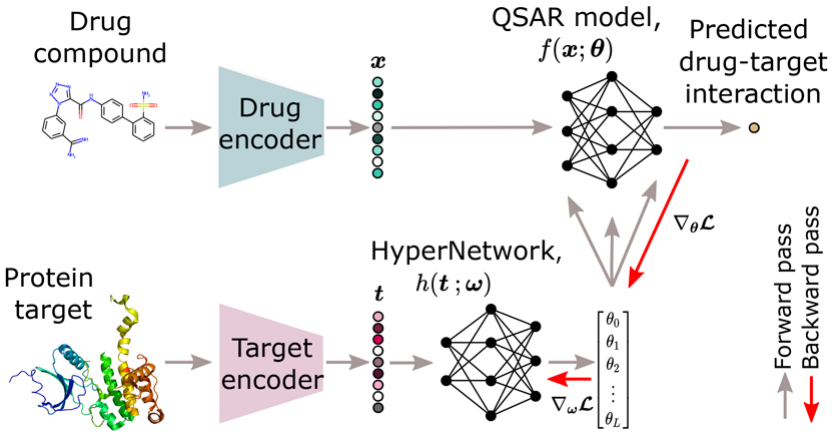

A central problem in drug discovery is to identify the interactions between drug-like compounds and protein targets. Over the past few decades, various quantitative structure–activity relationship (QSAR) and proteo-chemometric (PCM) approaches have been developed to model and predict these interactions. While QSAR approaches solely utilize representations of the drug compound, PCM methods incorporate both representations of the protein target and the drug compound, enabling them to achieve above-chance predictive accuracy on previously unseen protein targets. Both QSAR and PCM approaches have recently been improved by machine learning and deep neural networks, that allow the development of drug–target interaction prediction models from measurement data. However, deep neural networks typically require large amounts of training data and cannot robustly adapt to new tasks, such as predicting interaction for unseen protein targets at inference time. In this work, we propose to use HyperNetworks to efficiently transfer information between tasks during inference and thus to accurately predict drug–target interactions on unseen protein targets. Our HyperPCM method reaches state-of-the-art performance compared to previous methods on multiple well-known benchmarks, including Davis, DUD-E, and a ChEMBL derived data set, and particularly excels at zero-shot inference involving unseen protein targets. Our method, as well as reproducible data preparation, is available at https://github.com/ml-jku/hyper-dti.

## Introduction

Modeling of *drug–target interactions*(1) is a fundamental step of drug discovery that means learning the binding properties of interactions between small, drug-like compounds, hereby referred to as *drug compounds* (the term *drug compound* is used in this work to denote a ligand, i.e., molecular compound, considered as potential drug but that might not ultimately be appropriate as a final drug candidate), and the identified *protein target* of a disease.^2^ With the rise of new diseases, there is a strong need for fast and accurate prediction of a large number of drug–target interactions.^3^ In silico modeling of drug–target interactions benefit from a greater computational power^4^ and the use of state-of-the-art deep learning methods from other fields contribute further advancements.^5,6^ So far, research in this field has largely focused on the representations of the drug compound and the protein target, respectively.

Traditional models of quantitative structure–activity relationships (QSAR)^7^ learn the properties of drug–target interactions solely based on a representation of the structure of the drug compound. Commonly, the structure is first encoded into a numerical vector, i.e. embedding. Handcrafted embeddings can be constructed from chemical and biological data^8−10^ or through bit-vectors such as Morgan fingerprints.^11^ Alongside advancements in Natural Language Processing (NLP), also string representations of drug compounds, such as SMILES,^12^ can be processed with neural networks, either by using Recurrent Neural Networks (RNNs) with Long Short-Term Memory (LSTM),^13,14^ Convolutional Neural Networks (CNNs),^15^ or Transformers.^16−18^ Furthermore, graphs and point clouds provide higher dimensional representation that can be processed with Graph Neural Networks (GNNs).^19−21^ A crucial drawback with straightforward QSAR modeling of drug–target interactions is that predictions can never be made for new protein targets that the model has not been trained on, so-called *unseen* protein targets.^22^

As an alternative to QSAR models, Lapinsh et al.^23^ introduced *proteo-chemometric modeling (PCM)* to incorporate information about the protein target in the modeling. For these models, the protein target is first encoded to an embedding. Most commonly, NLP methods are applied to the amino acid sequence of the protein target,^24−26^ but the distance matrix specifying the contact map between each pair of amino acid in the protein target can also be used.^19,27^ Once both the drug compound and the protein target have been encoded, the modeling becomes a *multimodal* problem as it depends on inputs from multiple sources.^28^ The respective embeddings are then fused together, e.g. through concatenation, before further modeling of the drug–target interactions. Previous work have modeled concatenated embeddings with gradient boosting,^10,29^ and fully connected networks (FCNs).^15,30^ Other architectures include attention networks with various fusion mechanisms.^14,31^ The main advance over QSAR methods is that these models in principle allow to make better-than-random predictions for previously unseen protein targets, that is, protein targets for which no measured interactions with drug compounds are available. Such prediction tasks are extremely challenging even for powerful machine learning methods as well as traditional docking-based methods that utilize full 3D complexes of the drug compound inside a binding pocket of the protein target.^32^

**Figure 1:**
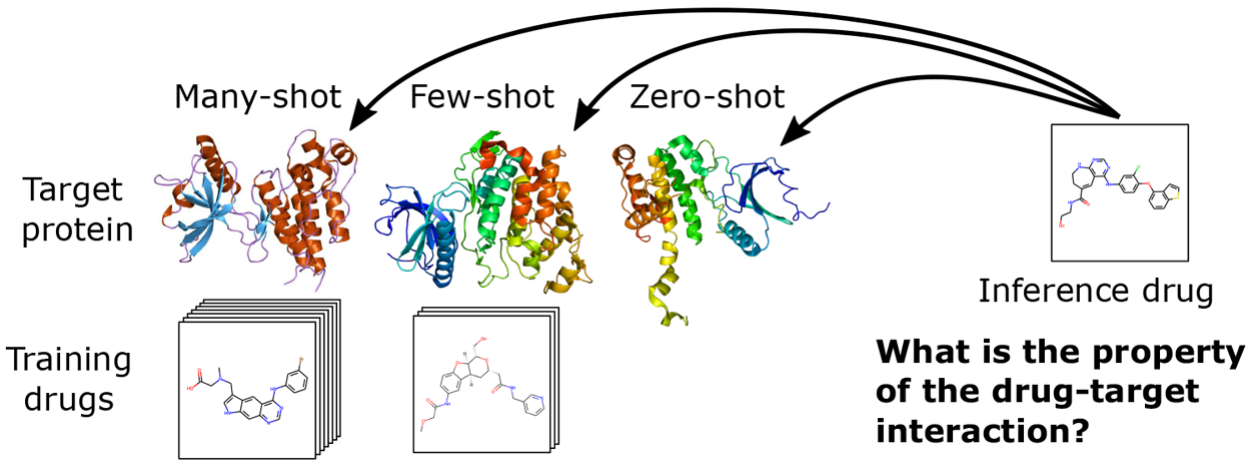
Problem setting, illustrating many-, few-, and zero-shot inference, based on the number of other drug–target interactions seen for the given protein target during training.

In the machine learning community, prediction tasks are distinguished based on the amount of training data available for the given task. Inference on a task for which sufficient data was available during training can be considered many-shot. Few-shot learning has emerged with techniques to train on tasks where only a few data points, usually less than 10, are available per task during the training process.^33−35^ By making use of available information about a given task, machine learning models are now able to make predictions during inference on tasks where no training data is available. Such inference is called *zero-shot*.^36^ During the prediction of drug–target interactions, the protein targets corresponds to the different tasks of the problem. Figure 1 illustrates the difference between many-, few-, and zero-shot inference when modeling drug–target interactions. Zero-shot performance can be evaluated by holding out certain protein targets for evaluation and thus running inference on unseen protein targets.

As deep neural networks are the dominant machine learning approach for all types of modeling involving molecules, they could also be candidates for PCM.^9,15^ However, deep neural networks require large amounts of training data and benefit from being trained on multiple tasks at once, through so-called *multitask learning*, where learned knowledge can be transferred between tasks. The same conclusion has been found for models that predict properties of drug–target interactions, namely that training simultaneously on multiple protein targets improves the overall performance.^37,38^ Due to the expensive and time-consuming experiments required to analyze drug–target interactions in a lab, there is typically a low amount of data available in total which creates a *low-data* problem.^33^ While PCM has enabled zero-shot inference of drug–target interactions,^9,15^ the setting is still challenging for the state-of-the-art models due to the shortage of data.^30^ In this work, we are aiming to both exploit the capabilities of deep neural networks to learn representations while at the same time forgoing their need for large amounts of data by efficiently transferring knowledge between tasks.

Recently, a special type of neural network called a *HyperNetwork*(39−41) is being used to tackle multitask learning for zero-shot inference in low-data settings. The broad definition of a HyperNetwork is that it is a neural network that in turn predicts the parameters of another neural network. Originally, Schmidhuber^39^ proposed fast weight adaptation as an alternative to Recurrent Neural Networks (RNNs). Since then, direct prediction of parameters has become the established method, first as dynamic convolutions^40^ and later under the name of HyperNetworks.^41^ The idea of HyperNetworks has been applied to a wide range of problems including neural architecture search,^42−44^ continual learning,^45^ differentiable pruning,^46^ image-based few-shot learning,^47^ and Bayesian inference in neural networks.^48^ Additionally, some methods of meta-learning can be understood as HyperNetworks.^49^ Knyazev et al.^44^ successfully use a HyperNetwork to predict the parameters of unseen architectures for CNNs, able to reach a remarkable performance of 60% accuracy on ImageNet. In this case, the HyperNetwork is a GNN that takes the computational graph of the CNN as input and directly outputs parameters for the CNN without any further training. Several other applications suggest that HyperNetworks equip the predicted neural networks with improved adaptation capabilities.^50,51^

Furthermore, HyperNetworks have been particularly successful in transferring information between tasks in multitask learning when the predicted parameters are conditioned on information about the given task.^52−58^ The neural networks predicted by such a HyperNetwork are referred to as *task-conditioned*. In personalized federated learning, Shamsian et al.^53^ predict client models using a HyperNetwork based on information about the client, i.e., task. While previous multitask multilingual models for NLP can suffer catastrophic forgetting,^59^ task-conditioned adaptors for the models predicted by a HyperNetwork better sustain the learned knowledge.^54,57^ Similarly, Zhmoginov et al.^56^ show that task-conditioned small CNNs predicted by their HyperTransformer improve generalization in few-shot learning. Other applications include a HyperNetwork semantic encoder that predicts parameters for a classifier^55^ and task-conditioned prediction of parameters in a healthcare setting.^58^ Overall, the mentioned research further motivates the use of HyperNetworks as a promising method for improving the generalization capabilities of neural networks.^49,54,58^

There are several more appealing properties of HyperNetworks that make them prime candidates for PCM. On a fundamental level, multiplication is the operation performed inside the neural network to the predicted parameters from the HyperNetwork, known as *multiplicative interactions*.^60^ Jayakumar et al.^60^ show that multiplicative interactions impose an inductive bias that strictly enriches the representation of neural networks. The result is shown to be particularly prominent during multimodal learning^61^ and for conditional computations.^62^ The task-conditioned parameters predicted by a HyperNetwork are typically distributed across multiple layers of the other neural network. As such, inference through the resulting neural network performs a higher number of multiplicative interactions and thus achieves greater sharing of information between the different input sources compared to more straightforward multimodal neural networks.^63,64^ Additional motivation for the improved generalization seen with the task-conditioned prediction of parameters using HyperNetworks, can be inferred from the theory provided by Lugosi and Neu.^65^ Their work shows that the bounds of the error in generalization between predicted and empirical results of supervised learning algorithms during inference, is related to the mutual information between the parameters of the neural network and the training data. The derived relation is that a lower dependency of the neural networks parameters on the training data puts a more strict bound on the error in generalization during inference. Therefore, the generalization capabilities of a HyperNetwork trained solely on information about the tasks are expected to be strictly equal to or greater than that of the more straightforward multimodal neural networks.

HyperNetworks have already been used for molecular property prediction.^66^ This neural network architecture achieved state-of-the-art performance on a number of benchmark data sets. However, no information about the protein targets was used and as such the prediction of parameters could not be task-conditioned nor could the resulting model perform zero-shot inference. Zhang et al.^36^ also propose the use of a HyperNetwork architecture in a drug discovery application, but rather for the prediction of drug synergy in cell lines of cancer patients. Their proposed HyperNetwork is designed for few-shot learning on cell lines with low amounts of data and can be understood as a task-conditioned prediction of parameters for the drug synergy model. State-of-the-art performance is achieved both for few-shot and zero-shot inference. We believe that by using embeddings of the protein targets for task-conditioned prediction of parameters using a HyperNetwork, a greater and more robust generalization to unseen protein targets can be reached when predicting binding properties of drug–target interactions.

In this work, we propose HyperPCM, a novel neural network architecture that addresses the challenges of predicting drug–target interactions in a low-data setting while enabling generalization to unseen protein targets. HyperPCM leverages the power of a HyperNetwork that learn to predict parameters for other neural networks, here acting as QSAR models. The success of our architecture is further attributed to two essential concepts borrowed from related work. First, we adopt a HyperNetwork-specific initialization strategy from Chang et al.^67^ that ensures stability in the predicted QSAR models. Second, we enrich the embeddings of the protein targets using the context module proposed by Schimunek et al.,^68^ which improves the robustness of HyperPCM. We evaluate HyperPCM’s effectiveness with extensive experiments on established benchmarks,^9,69,70^ comparing its ability against traditional machine learning baselines and prominent deep neural networks. The results demonstrate that HyperPCM achieves state-of-the-art performance in various settings including during zero-shot inference, where predictions are made for previously unseen protein targets. Additionally, our work contributes to the ongoing debate between sequence-based and structure-based modeling of drug–target interactions. On the Davis benchmark,^69^ we observe that language-based models outperform graph-based models, and on the DUD-E benchmark,^70^ the same language-based models outperform docking-based approaches that rely on full 3D structural information about the drug–target pair. Furthermore, HyperPCM offers a significant computational advantage for high-throughput screening applications and drug repurposing, pushing the boundaries of drug–target interaction prediction and making a valuable contribution to the field. Overall, our work presents a novel approach that combines HyperNetworks with QSAR modeling, offering a robust solution to the challenging problem of drug–target interaction prediction, especially in low-data settings.

## Methods

We propose HyperPCM, an architecture that uses a HyperNetwork *h* to predict task-conditioned parameters **θ** of another neural network *f*(***x***; **θ**) with input . The predicted parameters are task-conditioned as the HyperNetwork *h*(***t***; **ω**) relies on information about the task , e.g. a protein target, to output the full list of parameters **θ** for *f*. For applications in drug discovery, the method is a PCM approach and the predicted network can be considered a QSAR model. The set of tasks  contains the protein targets that can be represented by their amino acid sequences,^24,25^ or distance matrices.^19,27^ The set of inputs to the QSAR model  contains the potential drug compounds, represented as either SMILES strings,^13,16^ 2D molecular graphs,^20,71^ or 3D conformations.^72^ In both cases, the representations can be handcrafted or learned. Currently, unsupervised pretraining of encoders is a prominent approach to learning numerical embeddings. The most recent advancements come from contrastive methods.^72,73^

**Figure 2:**
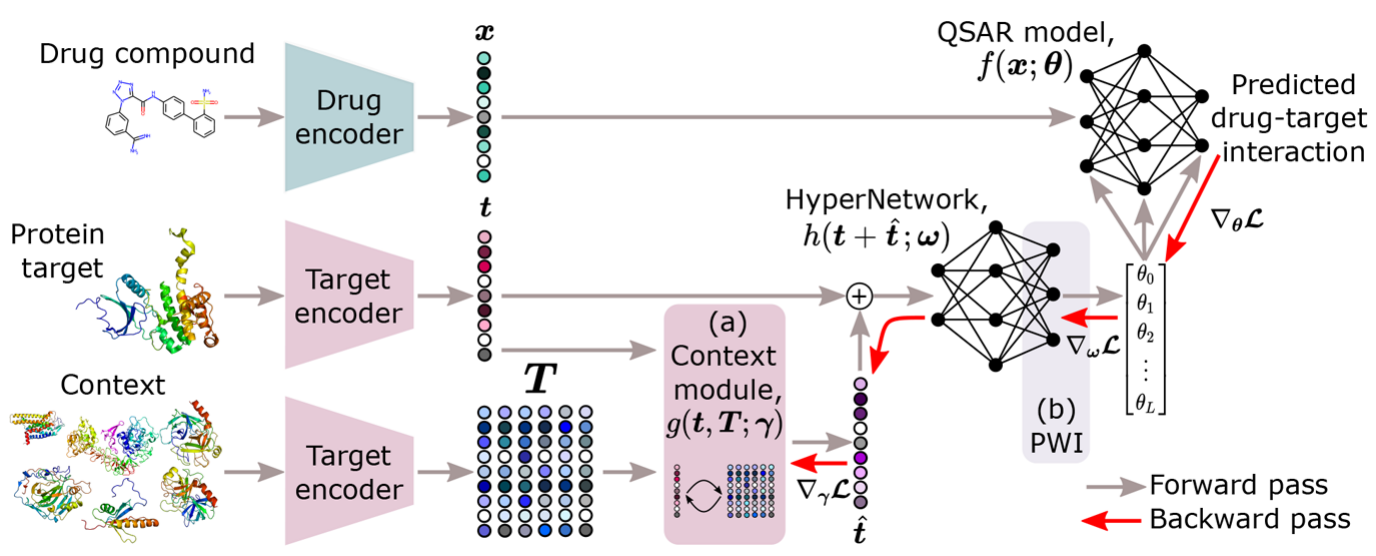
HyperPCM. Our approach to modeling drug–target interactions, in which a HyperNetwork predicts task-conditioned QSAR models based on (a) context-enriched embeddings of protein targets and with (b) the last layer of the HyperNetwork initialized through principled weight initialization (PWI).

The general formulation of the problem for a given set of training data  is defined as,
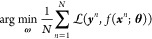
1
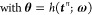
2where  is a differentiable loss function used to backpropagate the error of predictions versus the ground truth labels ***y*** using gradient descent. Due to the differentiable nature of all functions *f*, *h*, and , the system can be trained end-to-end. Figure 2 illustrates the overall setup of our proposed model, with the aim of modeling the binding properties of drug–target interactions. The architecture of the HyperNetwork depends on the nature of the representations used for the protein targets. When a pretrained encoder is used to first get a numerical embedding, the HyperNetwork and QSAR model can each be constructed as simple FCNs. Once trained, the HyperNetwork produces QSAR models that are specially meant to predict interactions between any drug compound and the protein target given to the HyperNetwork. The fact that each predicted QSAR model is solely concerned with predicting interactions toward a single protein target, allows it to be of comparably small size relative to ones trained for multitask applications. Additionally, Figure 2 shows (a) the context module that we use to enrich the embeddings of the protein targets and (b) the initialization strategy that we use in the last layer of the HyperNetwork to achieve stable inference in the QSAR model. The remainder of this section explains these two concepts.

### Context Module

Inspired by the idea that humans use associations to previously known information when they encounter unknown situations, HyperPCM comprises a context module^68,74^ to improve few-shot learning capacity. The context module uses a continuous Modern Hopfield Network (MHN)^75^ to associate a given sample pattern with a much larger external memory of known patterns, referred to as the context. Schimunek et al.^68^ originally proposed the context module as a means to enrich embeddings of drug compounds in a few-shot setting for molecular property prediction. In our method, the additional use of the information about the protein target allows us to explore the context module during zero-shot inference by enriching the embeddings of the protein targets rather than the ones for the drug compounds. As such, we train the MHN to retrieve a new protein target embedding from the larger context, that has amplified co-occurrences and covariate structures.^74^ While Schimunek et al.^68^ additionally use the context module to smooth out co-occurrences between query and support set samples, our application of the MHN only associates the given sample with the context. Prior work by Martin et al.^76^ exhibits similarities to our use of the context module. Their proposed Profile-QSAR model predicts binding affinity of drug compounds toward unseen kinase protein targets through a linear combination of properties from previously seen kinases using position-specific scoring matrices similar to those used in AlphaFold.^77^

In more detail, the MHN in the context module can be thought of as an associative memory that has similarities to the attention mechanism in Transformers.^78^ Given the context of all encoded unique protein targets from the training data  and the currently evaluated protein target embedding , the MHN retrieves an updated embedding according to
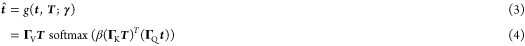
3where β is a scaling factor and **γ** is the set of trainable parameters in the MHN, including  with respective analogies to the query (Q), key (K), and value (V) concepts in Transformer’s attention mechanism.^75,79^ Note that *T* is the number of unique protein targets in the training set, not to be confused with *N* from eq 1, namely the total number of interactions in the training set. Like in previous work,^68,75^ we normalize the initial protein target embedding, context embeddings, and enriched embedding using LayerNorm.^80^ We use the full set of protein target embeddings from the training data as context, whereas Schimunek et al.^68^ rather sampled a subset of the training data for computational reasons.

### Signal Propagation

Initialization strategies have been introduced to ensure stable signal propagation through standard neural networks.^81−83^ A stable signal propagation means that the outputs retain the same distribution as their inputs. Under the assumption that the inputs to a linear layer ***z*** = ***Wx*** + ***b*** have *independent and identically distributed* (i.i.d.) features, such that , then LeCun et al.^81^ found that if  and *b*_*i*_ = 0 holds for all (i.i.d.) elements in ***W*** and ***b*** the distribution of output elements *z*_*i*_*∀i*, *j* follow
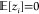
5
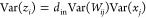
6where *d*_in_ is the input dimension, or *fan-in*, to the given layer. Expectations  and variances Var are taken across the random variables *x*_*j*_ ∼ *p*_data_ and *W*_*ij*_ ∼ *p*_**θ**_, which are independent at initialization. By scaling each weight element to , stable variance propagation can be achieved in the forward pass. To account for the effects of nonlinear activations, such as ReLU, the initial weights can be scaled by a factor .^83^

In the case of our HyperNetwork with a FCN architecture, we do not necessarily want to maintain variance as the outputs are themselves used as parameters in the other neural network. Throughout this section we refer to the other neural network as the *main network* following related work, but note that it is the same as the QSAR model in our case. Instead, the HyperNetwork should predict parameters that keep distributions invariant between the input and output of the main network. As such, Chang et al.^67^ propose *principled weight initialization (PWI)* that ensures predicted parameters start in a suitable range for each layer in the QSAR model.

Let  denote all layers of the HyperNetwork up to the last one and consider a HyperNetwork *head* to be a part of the last layer leading to a given subset of the output parameter vector **θ**. As such, we describe one of the HyperNetwork heads that predict parameters for ***W*** in the *l*^th^ layer of the main network as . Given that the aforementioned assumptions hold for all layers of the HyperNetwork, each output  should have zero mean and variance *c*_in_ Var(*H*_*i*′*k*_) Var(*t*_*k*_), where *c*_in_ is the input dimension to the last layer of the HyperNetwork. Note that we use *i*′ to refer to the entry representing the value for *W*_*ij*_. Because each output corresponds to a weight in the main network, the results can be directly plugged into eq 5*∀i*, *i*′, *j*, *k* to achieve
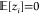
7
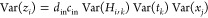
8Similarly, another HyperNetwork head can be used to predict the bias parameters ***b*** in the *l*th layer of the main network according to . Based on the same assumptions, these predicted parameters θ_*i*_^b^ have zero mean, but a variance of *c*_in_ Var(*G*_*ik*_) Var(*t*_*k*_). Due to the linearity of the variance, *∀i*, *i*′, *j*, *k* we get an increased variance for *z*_*i*_ in layer *l* of
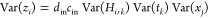
9
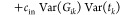
10Chang et al.^67^ use the result in eq 9 to conclude that a stable propagation in the main network, i.e. Var(*z*_*i*_) = Var(*x*_*j*_), can be achieved *∀i*, *i*′, *k* by setting
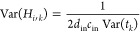
11and
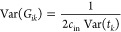
12which effectively shares the variance between weights and biases. Our HyperNetwork has separate heads for both weights and biases of each layer in the main network, all of which follow PWI.

## Experiments

We benchmark our proposed model HyperPCM on three data sets covering varying aspects and applications of drug–target interactions, including bioactivity, kinase inhibition, and molecular docking. On each benchmark, we compare our method against the, to our knowledge, currently best method by reproducing their evaluation scheme including splitting strategies and performance metrics. Additionally, we include other methods evaluated in comparable settings as well as our own baselines where possible. Methods that use more information than the sequences or structure of the drug compounds and protein target respectively, such as the location of the binding site,^84^ have been excluded from consideration unless directly comparable in other related work. We consider such methodologies out of scope for this work, as they rely on additional information. Table 1 gives an overview of the statistics of the three benchmarks.

**Table 1:** Overview of Benchmarks

Benchmark	#Interactions	#Drugs	#Targets	#Actives	#Inactives	Sparsity
Lenselink^9^^,^^a^	314,707	204,018	1,226	173,562 (55.15%)	141,145 (44.85%)	0.13%
Davis^69^^,^^a^^,^^b^	30,056	68	379	2,457 (8.17%)	27,599 (91.83%)	100%
DUD-E^70^	1,434,019	1,200,966	102	22,805 (1.59%)	1,411,214 (98.41%)	1.17%

a Actives/Inactives in the continuous benchmarks are based on the proposed thresholds: 6.5 for Lenselink, and 7 for Davis.

b Davis et al.^69^ state that the data set contains 442 kinase assays and 72 inhibitors, but this includes duplicated unique protein targets and noncanonical SMILES of the drug compounds that have been filtered out by Nguyen et al.^31^

The Lenselink benchmark is a balanced data set for the classification of active versus inactive drug–target interactions, i.e. bioactivity, derived from the ChEMBL database (version 20)^85^ in Lenselink et al.^9^ The balanced distribution of bioactivities in the Lenselink benchmark is imposed by the threshold used to separate the two classes and stems from the nature of the ChEMBL database. Namely, that there is a bias toward publishing active interaction. In reality, a drug–target interaction is far more likely to be inactive or nonbinding. Therefore, models trained on data sets such as the Lenselink benchmark might not transfer well to drug discovery projects in pharmaceutical companies, which contain a higher number of negative interactions. Combating this inherent bias in drug–target interaction data is an open question in the field.^86^ A naive strategy to increase the imbalance is to use all unobserved interactions as inactive, but this has the potential to introduce false negatives to the data while also resulting in too many data points for most modeling techniques.

The remaining two benchmarks explored in this work exhibit more realistic data distributions, achieved with other mechanisms. The Davis benchmark proposed by Davis et al.^69^ is a small data set, concentrated on interactions with kinase protein targets. It is modeled with continuous values for regression of binding affinity but has a skewed distribution toward lower affinities and, based on the proposed threshold, includes 8.17% active inhibitors.

Lastly, we include a benchmark commonly used to evaluate methods for molecular docking, called the Database of Useful Decoys: Enhanced (DUD-E).^70^ In DUD-E the imbalance has been manually constructed by generating 50 decoy drug compounds for every drug compound known to bind to a given protein target. The decoys were chosen to have physio-chemical properties similar to the active one but dissimilar typologies. Contrary to the Lenselink and Davis benchmarks, DUD-E only provides the binary labels for binding or nonbinding interactions without continuous affinity scores and thus can only be modeled as classification.

### Data Splitting

The evaluation is performed in various settings for each benchmark following strategies used in previous work on the respective data sets. Overall, the strategies include a fully *random* setting as well as different ways of holding out unique drug compounds and/or unique protein targets. Each hold-out strategy divides the available unique structures of drug compounds and/or protein targets along with all corresponding labeled interactions into separate folds. In turn, the resulting folds are used for training, validation, and testing respectively such that inference is only done on structures that were not seen by the model during training. Apart from a random setting, the evaluation on the Lenselink data set includes two ways of holding out unique drug compounds. First, based on the *temporal* entry of each unique drug compounds as in Lenselink et al.,^9^ and second based on a *k*-means (*k* = 10) clustering of 256-bit Morgan fingerprints^11^ representations of the drug compounds. The latter is referred to as *leave-cluster-compound-out (LCCO)* as proposed in Kim et al.,^30^ and it results in roughly equal-sized clusters in terms of number of labeled interactions. Additionally, the Lenselink evaluation includes a setting that holds out unique protein targets randomly, referred to as *leave-protein–out (LPO)* in Kim et al.^30^ For the Davis benchmark, we follow the strategies proposed by Nguyen et al.^31^ Unique drug compounds are held out randomly in the *cold-drug* setting, unique protein targets are held out randomly in the *cold-target* setting, and unique drug compounds and protein targets are held out randomly in the setting referred to as *cold*. Finally, for the DUD-E benchmark we follow the strategy used by Zheng et al.,^27^ Ragoza et al.,^87^ and Li et al.^88^ It follows a single splitting strategy that holds out unique protein targets based on a hierarchical clustering of similarity such that protein targets from the same protein family are kept in the same fold. In detail, the hierarchical clustering ensures that all proteins within a fold has ≥80% sequence identity.

Cross-validation is performed as in referenced work, using ten fixed folds for each Lenselink partition, and the three predefined folds of the DUD-E data set. For the Davis benchmark we use the predefined partitions proposed by ref (31), including a single fold for each splitting strategy. In order to assess variability, we instead make five reruns with varying random seeds for each splitting strategy. Similarly, in the temporal setting of the Lenselink benchmark cross-validation is not appropriate, instead ten reruns are made with new random seeds. In all cases, separate validation and test sets are created using the respective splitting strategies. Validation sets are used for model selection and test sets for final evaluation. All settings that hold out unique protein targets can be considered evaluating the performance of the models on zero-shot inference, as they test the model solely on protein targets for which no training data was available.

### Encoders

The HyperPCM model aims to be independent of the representations used for the drug compounds and protein targets, respectively. As such, we consider only pretrained, open-source encoders. In order to evaluate solely the novelty of our architecture, we also focus on the encoders from the baseline models without further fine-tuning or adaption to demonstrate that the performance improvement stems from our methodological approach. Kim et al.^30^ considered two language models pretrained on a large set of drug compounds to encode their SMILES strings, the recurrent autoencoder Continuous and Data Driven Descriptors (CDDD) from Winter et al.^13^ and the Transformer model MolBERT from Fabian et al.^16^ Regarding encoding of the protein targets, Kim et al.^30^ evaluated two other language models trained on the amino acid sequences of a large set of protein targets, UniRep^24^ and SeqVec.^25^ We extend the evaluation to also include two Transformer-based architectures for protein encoding, the ProtBERT and ProtT5 models,^26^ recently collected in the bio_embedding framework.^89^ Stärk et al.^90^ provide a more in-depth analysis and comparison between these encoders as well as the even bigger model ESM-1b.^17^ However, we exclude the use of the more recent ESM-2 model^18^ as it relies on a fixed sequence length. Based on the result in previous work, CDDD and SeqVec are primarily used in the HyperPCM setup as they gave the best performance during zero-shot inference in previous work.^30^

On the other benchmarks, Davis and DUD-E, the most prominent methods all use encoders trained end-to-end and thus are not considered for the HyperPCM model.^31,88^ Furthermore, note that some of the considered previous methods on these benchmarks make use of additional information, such as the distance matrix of the protein structure,^19,27,88,91^ the full 3D structures of the docked complexes of the drug compounds and protein target^87,92^ or information about the interaction site, i.e., the binding pocket.^71^

### Training Details

The HyperPCM model is implemented in PyTorch,^93^ and the predicted parameters are consistently distributed into the neural network of the QSAR model through reshaping. Backpropagation is done end-to-end using the Adam optimizer.^94^ A decaying learning rate, based on plateauing validation loss, is employed and models are trained until convergence with early stopping in terms of validation performance between predicted and ground-truth activity. The architectural decisions regarding the FCN making up the HyperNetwork as well as the QSAR model, such as the number of layers, the size of the hidden dimension, and the dropout rate are considered hyperparameters optimized as part of the model selection for each benchmark.

Batching of the data is nontrivial in a HyperNetwork learning setup. Inspired by the procedure from Knyazev et al.,^44^ we sample batches of both protein targets, referred to as *meta-batches*, and of drug compounds, referred to as *mini-batches*, respectively. However, additional measures are needed due to the large variation in drug compounds paired with unique protein targets. We perform an oversampling of underrepresented protein targets to enforce a minimum number of sampled drug compounds. We also sample a fixed number of drug compounds per protein target, by sampling with replacement in the rare cases that fewer drug compounds are available. Note that these sampling strategies only apply during the loading of training data, so as to not disturb the data distribution during inference. Varying batch sizes were explored as part of the model selection, independently for the meta- and mini-batches.

Further details about our model selection and hyperparameter optimization can be found in Table S1 of the Supporting Information.

### Loss Function

While the current state-of-the-art model on the Lenselink benchmark from Kim et al.^30^ was trained using Binary Cross-Entropy (BCE) loss for the classification problem, we revert to training the models on the regression problem directly on the provided continuous values as was done in the original work by Lenselink et al.^9^ However, we use the L1 loss function, also known as the Mean Absolute Error (MAE), instead of the Mean Squared Error (MSE) as used in Lenselink et al.^9^ motivated in the Supporting Information with Figure S1. In order to compare results to the previous models, we apply the fixed threshold of 6.5 log affinity from the benchmark after the training solely for evaluation purposes. On the Davis benchmark, where the custom is already to train model on the regression problem we use MSE for consistency with related work. Regarding the DUD-E benchmark, only binary labels are provided and thus we train the model using BCE.

### Performance Metrics

Following previous work on the Lenselink benchmark, we evaluate the predictive performance according to the Matthews Correlation Coefficient (MCC) metric. MCC is a discretization of the Pearson correlation with binary variables, originally introduced for protein secondary structure prediction by Matthews.^95^ As such, the MCC spans [−1, 1], where 1 indicates a perfect correlation and 0 represents random performance. The score is widely used within the bioinformatics community and evaluates the global model quality.^96^ Additionally, we provide Area Under the ROC-Curve (AUC) for all models evaluated, as a reference for the broader machine learning community.

For the regression problem posed in the Davis benchmark, all models are evaluated in terms of MSE and Concordance Index (CI) following previous work. The CI measures if the order of the predicted values *b*_*i*_, *b*_*j*_ of two random pairs of drug compounds and protein targets correspond to the order of their respective true values δ_*i*_, δ_*j*_.^97^ The metric is calculated as
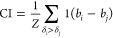
13where *Z* is a normalization constant and 1(*x*) is the step function, and we use the implementation from Nguyen et al.^20^

Evaluation on the DUD-E benchmark is done mainly using the AUC score. Furthermore, we evaluate the early enrichment of the model, as in prior work suggested important for drug discovery applications.^27,87,88^ Specifically, the ROC enrichment (RE) proposed by Jain and Nicholls^98^ as well as Nicholls^99^ is used, where the ratio between the true positive rate and the false positive rate is given in terms of varying thresholds. The RE for thresholds 0.5%, 1%, 2%, and 5% are evaluated. A random model achieves an RE score of 1.0 and the maximum score possible varies depending on the threshold, for example being 20.0 for the 5% RE score.^87^

## Classification of Bioactivity

Previous work on the Lenselink benchmark has produced a number of baselines with traditional machine learning and deep learning methods on concatenated embeddings of the drug compounds and protein targets. Picking out the leading results, we compare our novel method to two deep FCNs, DNN_PCM,^9^ and DeepPCM,^30^ applied to handcrafted as well as unsupervised, learned embeddings. Also, from Lenselink et al.^9^ we include a random forest multiclass model. Note that the handcrafted embedding used with DNN_PCM and the random forest model by Lenselink et al.^9^ include physio-chemical descriptors such as molecular weight and lipophilicity, commonly used in traditional QSAR modeling. The results using handcrafted embeddings are reiterated from the respective reference work, but all other baselines are reimplemented and rerun on our new splits. Table 2 presents average MCC scores across the ten test sets of the 10-fold cross-validation in the random, LCCO, and LPO settings, or reruns in the temporal case. The highest performance, from either referenced work or our reruns, is presented with improved results marked as reimplemented. Tables S3 and S4 in the Supporting Information present the full result from only our reimplementations, both in terms of MCC and AUC. Additionally, we include a gradient boosting baseline namely XGBoost,^100^ see Table S2 in the Supporting Information for details on the hyperparameters. Our method HyperPCM achieves the highest average score in each setting, marked in bold. Out of the three benchmarks included in this work, Lenselink includes a greater variety of unique protein targets. However, there is no lower limit to how many interactions are available per protein target. In fact, in the Lenselink benchmark the number of known interaction per protein target varies between a single one and over 4000, which makes it a particularly challenging problem for learning to transfer knowledge between protein targets.

**Table 2:** Lenselink Benchmark^a^

	Embedding		Few-/many-shot			Zero-shot
Model	Drug	Target	Random	Temporal	LCCO	LPO
RF^9^	Handcrafted		0.670	0.210	N/A	N/A
DNN_PCM^9^	Handcrafted		0.610	0.330	N/A	N/A
DeepPCM^30^	MolBERT	UniRep	0.654 ± 0.005	*0.370 ± 0.008*^b^	*0.505 ± 0.053*	0.312 ± 0.024
	MolBERT	ProtBERT	0.625 ± 0.006^b^	0.362 ± 0.006^b^	0.455 ± 0.054^b^	0.299 ± 0.043^b^
	MolBERT	ProtT5	0.620 ± 0.004^b^	0.360 ± 0.003^b^	0.452 ± 0.057^b^	0.296 ± 0.040^b^
	MolBERT	SeqVec	0.639 ± 0.003^b^	*0.370 ± 0.006*^b^	0.487 ± 0.062	0.311 ± 0.035
	CDDD	UniRep	0.630 ± 0.008	0.352 ± 0.009^b^	0.490 ± 0.061^b^	0.307 ± 0.031
	CDDD	ProtBERT	0.635 ± 0.004^b^	0.343 ± 0.006^b^	0.462 ± 0.060^b^	0.294 ± 0.049^b^
	CDDD	ProtT5	0.634 ± 0.005^b^	0.353 ± 0.006^b^	0.460 ± 0.056^b^	0.310 ± 0.054^b^
	CDDD	SeqVec	0.643 ± 0.005^b^	0.363 ± 0.006	0.478 ± 0.048^b^	*0.322 ± 0.028*
XGBoost	CDDD	SeqVec	0.510 ± 0.005	0.366	0.408 ± 0.043	0.304 ± 0.040
HyperPCM	CDDD	SeqVec	**0.682 ± 0.039**	**0.395 ± 0.005**	**0.532 ± 0.059**	**0.340 ± 0.051**

a Average MCC over 10-fold cross-validation in the random, LCCO, and LPO settings and ten re-runs in the temporal setting. The best performance per setting is marked in bold and the best-performing baseline is marked in italic. Underlined HyperPCM results illustrate statistically significant (*p* < 0.05, paired or unpaired depending on identical splits or not) improvements over the best baseline excluding the random setting in which statistical significance over the single RF run cannot be evaluated. However, note that HyperPCM significantly outperforms the re-implemented DeepPCM model with identical encoders and splits across all settings, see Tables S3 and S4 in the Supporting Information. Results marked N/A are not available.

b Reimplemented.

We test the statistical significance of our model’s improvement over previous methods, by performing paired Wilcoxon signed-rank tests where applicable. The alternative hypothesis is that our model’s mean MCC score is greater than the score of (1) the best-performing baselines from Table 2, and (2) the most similar baseline DeepPCM using the same encoders, CDDD and SeqVec. For the cases when the original result from Kim et al.^30^ was higher than our reimplementation (not referenced as reimplemented in Table 2), we instead ran unpaired, Wilcoxon rank-sum tests because of the nonidentical cross-validation splits.

In the random setting, none of the previous deep learning methods beat the random forest model. Our method on the other hand does improve slightly over the random forest model, although significance cannot be tested as only a single run is presented in Lenselink et al.^9^ When compared to the DeepPCM model using the same encoders, the improvement is more significant (*p* = 0.010, paired). Similarly, in the LCCO cross-validation our model significantly outperforms the most similar baseline (*p* = 0.001, paired) while only seemingly improving over the best-performing baseline (*p* = 0.113, unpaired). The results in the temporal setting vary less as they present reruns on a single test set and our model significantly improves over all included baselines with *p* < 0.001 (paired) for all cases. In the zero-shot setting our model marginally improves over the most similar and best-performing baseline, but the result is not significant (*p* = 0.224, unpaired). While MCC scores below 0.5 might not be considered practically useful in some cases, the overall achievement is still a strong enrichment over otherwise random prediction with scores around 0.

Nevertheless, it is important to note that the statistical tests are not entirely fair in the cases where we could not reproduce the results presented in Kim et al.,^30^ e.g., DeepPCM with MolBERT and UniRep in the LCCO setting and DeepPCM with CDDD and SeqVec in the LPO setting. In these cases, the data splits are not identical, as the exact data splits used in Kim et al.^30^ were not documented. We split the data using the same procure described in the previous work.^30^ Our own reimplementation of DeepPCM with CDDD and SeqVec achieve an MCC score of 0.316 ± 0.043 in the LPO setting, which is still higher than all other baselines, and the improvement over this result with our HyperPCM model can be considered statistically significant (*p* = 0.024, paired). In the Supporting Information and Figure S2, we present an extended analysis of a subset of the LPO split where only drug compounds that were not seen during training are used in the test sets. The performance of our method in the extended setting is 0.313 ± 0.058 compared to 0.281 ± 0.051 for the best baseline, which also is a significant improvement (*p* = 0.002, paired).

### Ablation Study

**Figure 3:**
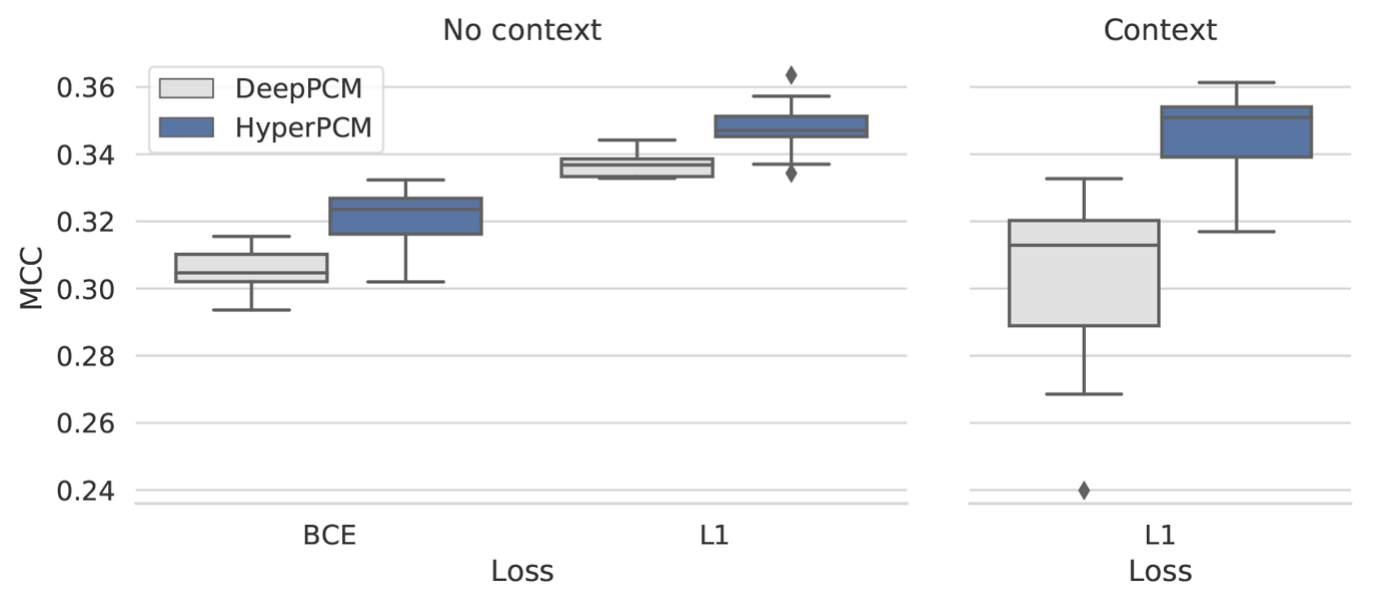
Ablation study. Comparing the baseline DeepPCM in two different training settings and the additional improvement with our proposed HyperNetwork setup, with or without context-enriched protein embeddings. Showing MCC across ten reruns on a fixed data split.

Further, we conduct an ablation study to analyze the importance of each of our three methodological contributions. Figure 3 presents the performance in terms of MCC on a fixed train/test split from the zero-shot setting, over ten reruns with different random seeds. Both the baseline DeepPCM and our model HyperPCM respectively achieve improved performance when training with L1 loss compared to when trained on the binary active/inactive labels, as done in the previous work.^30^ The learning curves shown in Figure S3 of the Supporting Information provides a possible explanation for the trend, namely that both models largely overfit when trained with BCE. Moreover, Figure 3 shows that performance gains are generally achieved with our proposed HyperNetwork setup, both when trained for classification and regression. Lastly, the rightmost panel of Figure 3 illustrates the effect of adding the context module to enrich the embeddings for the protein targets in the baseline model versus in our HyperNetwork model. The increased variance in performance should be explored further in future work. Regardless, the average results are slightly increased for the HyperNetwork setup when context-enriched embeddings are used.

### Computational Cost

An important drawback with our proposed HyperNetwork architecture is the increased computational cost of the training procedure. The complexity arises from the large size of the HyperNetwork that is needed in order to predict all parameters of the QSAR model. The average computational cost in terms of GPU hours for training and evaluating the HyperPCM model on the Lenselink benchmark is 54.67 ± 34.38 in the random split, 10.74 ± 5.75 in the temporal split, 15.51 ± 3.81 in the LCCO split, and 7.63 ± 4.65 in the LPO split. A significant increase compared to the computational cost for our reimplementation of the DeepPCM that is 0.24 ± 0.06 in the random split, 0.17 ± 0.05 in the temporal split, 0.18 ± 0.05 in the LCCO split, and 0.53 ± 0.27 in the LPO split. The advantage on the other hand is the comparably smaller size of the QSAR model produced by our trained HyperPCM model compared to the need to run the full DeepPCM model for inference on any drug–target interaction pair.

## Drug–Target Binding Affinity Prediction

An extensive list of methods has been evaluated in the random setting of the Davis^69^ benchmark, following the cross-validation procedure of Öztürk et al.^15^ However, a smaller subset of the models has been evaluated in the more challenging case of zero-shot inference. We compare our novel method to models evaluated precisely according to the strategies described in Nguyen et al.^31^ on the Davis data set. Additionally, we provide a more extensive comparison in the random setting of the Davis benchmark in Table S6 of the Supporting Information. All previous models considered, use a molecular graph representation for the drug compounds, processed using different Graph Neural Networks (GNNs). The protein targets are represented as amino acid sequences processed with a CNN in GraphDTA^20^ and the TAPE^101^ encoder in the models proposed by Nguyen et al.^31^ DGraphDTA^19^ on the other hand uses the contact map of each protein target to generate a graph, in turn processed with a GNN. The GraphDTA and DGraphDTA models further use a deep FCN for the concatenated embeddings, whereas Nguyen et al.^31^ propose two fusion networks, Graph Early Fusion Affinity (GEFA) and Graph Late Fusion Affinity (GLFA).

Table 3 presents the average CI scores across five reruns of the predefined test set proposed by Nguyen et al.^31^ in the random, cold-drug, cold-target, and cold settings. Complementary results in terms of MSE scores are also provided in Table S5 of the Supporting Information. Results for the previous models GraphDTA, DGraphDTA, GLFA, and GEFA are referenced from Nguyen et al.,^31^ where error bars were not provided. Additionally, we include two of our own baselines, a random forest and an XGBoost model implemented with the same encoders as the HyperPCM model and evaluated with five reruns. For the random forest, standard hyperparameters from scikit-learn^102^ were used, and for the XGBoost we adopted the hyperparameters as previously optimized on the Davis benchmark from Thafar et al.;^29^ see Table S2 of the Supporting Information for more details. The best average result for each setting is marked in bold. Analogously to the Lenselink experiments, we test the statistical significance of the results using paired Wilcoxon signed-rank tests. In this case we can also test the results of previous models as they where all evaluated on identical data partitions. No statistical significance (*p* < 0.05, paired) can be determined among the top performing models in either of the settings. The average computational cost in terms of GPU hours for training and evaluating the HyperPCM model on the Davis benchmark is 2.72 ± 0.38 in the random split, 1.35 ± 0.23 in the cold-drug split, 0.48 ± 0.09 in the cold-target split, and 0.13 ± 0.02 in the cold split.

**Table 3:** Davis Benchmark^a^

	Embedding		Few-/many-shot		Zero-shot	
Model	Drug	Target	Random	Cold-drug	Cold-target	Cold
GraphDTA^20^^,^^b^	GCN	CNN	0.865	0.678	0.729	0.578
GraphDTA^20^^,^^b^	GIN	CNN	0.879	0.676	0.707	0.627
DGraphDTA^19^^,^^b^	GCN	GCN	0.887	0.534	0.766	0.608
GLFA^31^^,^^b^	GCN	TAPE	**0.895**	0.670	0.780	0.636
GEFA^31^^,^^b^	GCN	TAPE	0.893	0.709	0.795	0.639
RF	CDDD	SeqVec	0.867 ± 0.001	0.700 ± 0.005	**0.805 ± 0.001**	0.679 ± 0.006
XGBoost	CDDD	SeqVec	0.890 ± 0.001	**0.713 ± 0.008**	0.802 ± 0.001	0.668 ± 0.007
HyperPCM	CDDD	SeqVec	0.894 ± 0.003	0.684 ± 0.014	0.795 ± 0.008	**0.690 ± 0.031**

a Average CI from five re-runs with varying random seeds. Standard deviation is displayed when available. The best performance per setting is marked in bold. Underlined results are statistically significant (*p* < 0.05, paired).

b Results repeated from Nguyen et al.^31^

As seen in Table 3, the top performing models on this benchmark differ between each of the four settings, and while no statistical significance can be found between the top performing models of each setting, there is still consistency in the improvements provided here over previous work on the benchmark. In the random setting, the graph-based fusion method GLFA achieves the best performance, in the cold-drug setting our XGBoost baseline, and in the cold-target setting the RandomForest baseline. Regarding the most difficult zero-shot setting, cold, HyperPCM achieves the highest CI, significantly outperforming all other models (*p* < 0.05, paired) except the random forest model (*p* = 0.313, paired) which also achieves a better score in terms of MSE. In both of the settings that tests zero-shot inference, cold-target and cold, most of the models using the string-based encoders significantly outperform the previous, graph-based models. Similar results have been observed in other studies on molecular representation learning, in which graph representations of drug compounds were found less beneficial than vector or string representations.^103−106^

There are a number of key differences between the Davis and Lenselink data sets that explain variations of the results seen in Table 3 compared to Table 2. All in all, Davis is significantly smaller in size with only around 26k labeled interactions compared to 315k labeled interactions in Lenselink. Additionally, the number of unique protein targets is fewer in Davis and they all belong to the same protein family, kinases. Even more extreme, is the very restricted number of drug compounds in the Davis benchmark containing only 68 unique canonical structures of drug compounds. On the other hand, the Davis data set is *dense* in the sense that all protein targets have labeled interactions with all drug compounds. As a result, the minimum number of data points for each protein target is always 68 whereas it can be as low as one for some protein targets in the Lenselink benchmark.

The low number of drug compounds explains the generally lower performance in the settings that test on unseen drug compounds (cold-drug and cold). Recall that these settings were generally easier for the models on Lenselink (temporal and LCCO), where a greater variety of data is available. However, the dense nature of the data set (see paragraph above) provides a possible reason as to why the tree-based methods are competitive with our HyperNetwork method. One of the benefits of the multiplicative interactions introduced in the task-conditioned prediction of parameters using a HyperNetwork, is to improve learning on low-data protein targets. For the Davis benchmark, the regular modeling of concatenated embeddings in the tree-based methods appears sufficient, as each protein target has a minimum of 68 data points in the training data. Similarly, our method is also designed to improve the performance when predicting interactions toward unseen protein targets. Indeed, the performance of HyperPCM is most competitive compared to other methods in the settings with held-out protein targets, cold-target and cold. Despite that, the fact that all protein targets in the benchmark are from the same family implies a greater similarity between all protein targets. Therefore, the HyperPCM model’s ability to better generalize between more diverse protein targets is also not fully utilized in the Davis benchmark.

In summary, the overall small size of Davis is manageable for the tree-based models which also perform better than all previous models evaluated on the benchmark. Given their relatively low computational cost, they are likely preferable in this case. The HyperPCM is instead more advantageous on larger data sets, where the computational cost of the tree-based methods grows, and where there is a greater variety in the properties of the protein targets and more low-data protein targets are included.

## Molecular Docking Benchmark DUD-E

In the field of molecular docking there are three types of tasks for computational methods: *pose prediction* of the drug compounds inside the binding pocket of the protein target, *virtual screening* to determine weather of not an interaction is binding, and *scoring* the binding affinity of the interaction.^98^ The DUD-E benchmark is commonly used to evaluate molecular docking methods due to the extensive information provided about the drug compounds and protein targets, including 3D structure as well as docking specific details such as binding site location.^70^

The current state-of-the-art methods in the virtual screening application of the DUD-E benchmark; DrugVQA,^27^ MINN-DTI,^88^ and GanDTI,^91^ can be understood as PCM approaches and are all evaluated as originally proposed by Ragoza et al.^87^ They process the drug compounds either as SMILES strings or molecular graphs, and the protein targets either as amino acid sequences or contact maps. All three models apply FCNs to concatenated embeddings of the drug compound and protein target of each interaction. However, contrary to previously mentioned PCM methods, they also include interconnection between the two encoders in different ways. Thus, there are more similarities between these models and our HyperPCM method, but neither of them can be understood as HyperNetworks. Earlier benchmarked models on the DUD-E data set have utilized more information about the drug–target interaction. First, PocketGCN^71^ is a PCM model where the binding pocket of the protein target is used as input, which requires the additional information specifying where this pocket is located. Other methods predict binding based on the 3D complexes of the drug compounds and protein target. This is the case for the deep learning-based methods 3D-CNN^87^ and GNN_DTI,^92^ the docking-based method AutoDock Vina,^32^ and the scoring-based traditional machine learning approaches, RF-score^107^ and NNScore.^108^

In Table 4, the average performance across three test sets of the 3-fold cross-validation are presented for our HyperPCM model and related methods. The results from all previous methods are taken from the respective original work, apart from the NNScore, RF-Score, and AutoDock Vina models that were evaluated by Ragoza et al.^87^ The average computational cost in terms of GPU hours for training and evaluating the HyperPCM model is 0.25 ± 0.04. All PCM approaches can be seen to greatly outperform the scoring-based and docking-based approaches across all performance metrics. Among the PCM approaches, HyperPCM exhibits competitive performance with GanDTI in all metrics, significantly outperforming it according to the lower-threshold RE scores. Given that the performances of HyperPCM and the top related methods are close to the maximum across all metrics, we believe that this is a fairly easy benchmark that can now be sufficiently solved with various PCM alternatives. The interesting take-away from the DUD-E benchmark is that even while utilizing simpler and more easy-to-come-by information about the drug–target pair, current deep learning methods greatly improves over more traditional approaches such as docking for the task of virtual screening.

**Table 4:** DUD-E Benchmark^a^

	Zero-shot				
Model	AUC	0.5% RE	1% RE	2% RE	5% RE
NNScore^108^	0.584	4.166	2.980	2.460	1.891
RF-Score^107^	0.622	5.628	4.274	3.499	2.678
AutoDock Vina^32^	0.716	9.139	7.321	5.811	4.444
3D-CNN^87^	0.868	42.559	29.654	19.363	10.710
PocketGCN^71^	0.886	44.406	29.748	19.408	10.735
GNN_DTI^92^	0.968	124.031	69.037	38.027	16.910
DrugVQA^27^	0.972 ± 0.003	88.17 ± 4.88	58.71 ± 2.74	35.06 ± 1.91	17.39 ± 0.94
MINN-DTI^88^	0.992 ± 0.007	175.89 ± 12.02	90.77 ± 5.81	46.49 ± 2.63	19.10 ± 0.71
GanDTI^91^	**0.997**	71.13	68.78	**49.40**	**19.79**
HyperPCM	0.995 ± 0.003	**190.02 ± 3.48**	**96.02 ± 1.63**	48.37 ± 0.76	19.55 ± 0.25

a Average performance from the 3-fold cross-validation originally proposed by Ragoza et al.,^87^ evaluated on held-out protein targets. Standard deviation displayed when available. The best performance per metric is marked in bold. Statistical significance cannot be determined among the best performing models; MINN-DTI, GanDTI, and HyperPCM. Results for the NNScore, RF-Score, and AutoDock Vina models are repeated from Ragoza et al.^87^ All other results are repeated from the respective references.

## Discussion and Conclusions

We have proposed HyperPCM, a HyperNetwork-based architecture that directly predicts task-conditioned parameters of a QSAR model for prediction of drug–target interactions. The multiplicative interactions of the architecture enhance its adaptability across diverse protein targets, even with limited training data. Moreover, the specialized weight initialization strategy of the HyperNetwork stabilizes the signal propagation through the QSAR model. By enriching the embeddings of the protein targets through an MHN in the HyperNetwork, the model’s robustness to unseen protein targets is improved. We demonstrate our architecture’s effectiveness on three established benchmarks, showcasing significant performance improvements across various scenarios including during zero-shot inference.

While HyperPCM can adjust to new proteins at test time, without the need to be retrained, one of its limitations is the high computational cost associated with the training process compared to previous work. Thus, on smaller data sets with higher per-target data availability and lower diversity between protein targets, tree-based methods might be preferred. However, HyperPCM exhibits clear advantages in scenarios demanding zero-shot inference on new protein targets. In this case, HyperPCM extends the expressive power of previous methods with more learnable parameters while decreasing the complexity of inference once the QSAR model has been predicted by the HyperNetwork. An example of such a scenario is when a new protein target has been identified for a given disease, but no experimental bioactivity data or binding affinity measurements are available. In this scenario, information from other proteins has to be carried over in order to perform meaningful virtual screening.^109^ Despite the fact that the performance of our model as well as prior work in the zero-shot setting of the Lenselink benchmark is not high enough to be practically useful yet, we believe that the contribution provides a stepping stone for future work.

A promising direction for future research on prediction of drug–target interactions, is to leverage recent advances in machine learning for representation learning of small molecules and proteins. The incorporation of contrastive pretraining methods, such as CLOOME,^73^ could improve the representations of the drug compounds or protein targets. Despite prior limitation with graph-based encoders of drug compounds, we also believe that the 3D structures of protein targets should be explored further. Recent advancements with Equivariant Graph Neural Networks (EGNN)^110^ are proving effective and might allow improved representations of 3D structures due to the remarkable progress achieved in protein structure prediction, driven by breakthroughs like AlphaFold.^77^ While docking-based methods already use the full 3D complexes of the drug–target pair, they are currently outperformed by PCM methods using solely sequence-based representations. Still, DGraphDTA,^19^ which models the 3D structure of the protein targets, showed competitive performance in the easier random setting of the Davis benchmark. This suggests that methods that perform well in more difficult low-data settings, such as our HyperPCM model, could benefit from similar representation of the protein targets.

Furthermore, microRNAs have been identified as critical targets in gene expression and disease progression as they hold the capacity to modulate intricate regulatory networks.^111^ As such, there is a strong need to understand their interactions with drug compounds. The challenges associated with experimental validation and the high-dimensional nature of the interactions makes the use of computational methods vital. Bounded Nuclear Norm Regularization^112^ and the Deep Autoencoder and Scalable Tree Boosting Model (DAESTB)^113^ are two useful methods that already exist for the prediction of drug–target interactions with microRNAs. Our belief is that HyperPCM, with its capacity for accurate prediction of drug–target interactions even in low-data settings, could effectively enhance the prediction of interactions between drug compounds and microRNAs.

## Data Availability

Python code and instructions to reproduce the predictive results of drug–target interaction predictions benchmarked on the Lenselink, Davis, and DUD-E data sets are available at https://github.com/ml-jku/hyper-dti under the MIT license. Instructions to prepare the programming environment, as well as to download the data and run the training, inference, and evaluation procedure can also be found there. The code was run on a cluster of servers of diverse Nvidia GPUs including Titan V (12GB), V100 (16GB), A10-100 (20–48GB), GTX (1080 Ti, 11GB), RTX (2080 Ti, 11 GB), Tesla P40 (24GB), and Tesla P100 (16GB) using PyTorch 1.9.1.

## Supporting Information Available

Additional details on training and hyperparameter optimization of HyperPCM as well as detailed hyperparameters of the two tree-based baselines Random Forest and XGBoost; an analysis of the Lenselink benchmark; complementary results for the bioactivity prediction on the Lenselink benchmark; and complementary results for the drug–target binding affinity prediction on the Davis benchmark (PDF)
