# Supplementary material for: HyperPCM: Robust Task-Conditioned Modeling of Drug–Target Interactions
Source: J Chem Inf Model. 2024 Jan 8;64(7):2539–53. doi: 10.1021/acs.jcim.3c01417 (PMC11005051; doi:10.1021/acs.jcim.3c01417)
Supplement: Supplementary file 1 — ci3c01417_si_001.pdf [file ci3c01417_si_001.pdf]

Supporting Information for HyperPCM: Robust Task-Conditioned Modeling of Drug-Target Interactions

Emma Svensson,[†,‡] Pieter-Jan Hoedt,[†] Sepp Hochreiter,[†,¶] and Günter Klambauer[*,†]

†*ELLIS Unit Linz & Institute for Machine Learning, Johannes Kepler University Linz, 4040 Austria*

‡*Molecular AI, Discovery Sciences, R&D, AstraZeneca, Gothenburg, 413 83 Sweden*

¶*Institute of Advanced Research in Artificial Intelligence (IARAI), Vienna, 1030 Austria*

E-mail: klambauer@ml.jku.at

This document provides details on training and hyperparameter selection, then an analysis of the Lenselink benchmark, complementary results for the bioactivity prediction on the Lenselink benchmark, as well as complementary results for the kinase inhibition prediction on the Davis benchmark.

Training and hyperparameter details

In the following supplementary material, we presents an overview of the considered hyperparameters from our model selection phase in Table S1, including the explored values and final options marked in bold. Note that the explored sizes of the mini-batches implicitly determines the degree of over-/undersampling done as described in our work. Increasing sizes of mini-batches means an increased number of oversampled drug compounds for protein targets that do not have enough labeled interactions. While by decreasing the mini-batch size an increased oversampling of protein targets is instead needed. For the most part the optimization from the Lenselink benchmark was used without further tuning to the Davis and DUD-E benchmarks. However, on the Lenelink benchmark the QSAR model re-used the white noise module from DeepPCM followed by a simple fully-connected layer of 512 units, whereas on the Davis and DUD-E benchmarks the noise module was removed and replaced by two fully-connected layers of size 512 and 256. Also, for the Lenselink benchmark the context module was followed by a skip connection as in Fig. 2 of the main paper, but this was removed for the Davis and DUD-E benchmarks. Due to the small size of the DUD-E benchmark, the learning rate had to be reduced to 0.0000005 and the weight decay increased to 0.00005 in order to combat the otherwise high degree of overfitting.

Furthermore, Table S2 provides a detailed list of the hyperparameters used in the random forest and XGBoost models respectively.

Table S1: Considered hyperparameter space for model selection, with selected configurations based on manual search on validation set shown in bold.

	Hyperparameter	Explored space
Training	Optimizer	{**Adam**}
	Learning rate	{0.00005, **0.0001**, 0.0005, 0.001}
	Scheduler	{None, **ReduceOnPlateu**}
	Weight decay	{**0.00001**, 0.0001, 0.001}
	Meta-batching (Targets)	{**32**, 128, 256, 512}
	Mini-batching (Drugs)	{**32**, 256, 512, Full}
HyperNetwork	Target encoder	{UniRep, **SeqVec**, ProtBERT, ProtT5}
	Number of hidden layers	{0, **1**, 2, 4}
	Hidden dimension	{16, 64, **256**, 512}
	Dropout	{0, **0.25**, 0.5, 0.75}
	Activation	{**ReLU**, SELU}
	LayerNorm	{**False**, True}
Context module	Hidden dimension (QK)	{256, **512**}
	Number of Hopfield heads	{4, **8**}
	Scaling factor, β	{0.1, 1/256, **1/512**}
	Dropout	{**0.5**}
QSAR model	Drug encoder	{MolBERT, **CDDD**}
	Number of hidden layers	{0, **1**, 2}
	Hidden dimension	{16, 64, 256, **512**}
	Dropout	{0, **0.25**}

Table S2: Hyperparameter used for the baselines. The random forest was trained using the standard settings in scikit-learn[1] whereas XGBoost was trained with the hyperparameters from Thafar et al.[2] as optimized on the Davis benchmark.

Hyperparameter	RandomForestRegressor	XGBoostRegressor
n_estimators	100	855
criterion/objective	squared_error	reg:squarederror
max_depth	None	19
min_sample_split	2	N/A
min_sample_leaf	1	N/A
min_weight_fraction_leaf	0.0	N/A
max_features	1.0	N/A
max_leaf_nodes	None	N/A
min_impurity_decrease	0.0	N/A
bootstrap	True	N/A
warm_start	False	N/A
ccp_alpha	0.0	N/A
max_samples	None	N/A
booster	N/A	gbtree
colsample_bytree	N/A	0.8
learning_rate	N/A	0.03
scale_pos_weight	N/A	1
gamma	N/A	0
alpha	N/A	5
tree_method	N/A	auto
min_child_weight	N/A	5

Analysis of the Lenselink benchmark

Lenselink et al.[3] proposed a benchmark dataset of drug-target interactions derived from the ChEMBL database. The dataset includes 204,017 drug compounds and 1,226 protein targets, together making up 314,707 experimentally tested interactions with labeled bioactivity in terms of log affinity values. Fig. S1 illustrates the distribution of labels in the full dataset. For the purpose of creating a balanced split into the two classes active or inactive, Lenselink et al.[3] imposed a fixed threshold of 6.5 log affinity on the bioactivities. The current state-of-the-art model on the Lenselink benchmark from Kim et al.[4] was trained using these binary labels. However, real-world data is not balanced, many more interactions between drug compounds and protein targets are considered to be inactive. The reason why the ChEMBL database contains a majority of active compounds is that it is based on literature data where compounds are more likely to be included in a publication if they are active. Skewed data in this way is a common problem in the field and for applications of machine learning approaches. As such, we believe it to be less beneficial to restrict the learning to these arbitrary class labels. Rather, it should be more informative for the models to make use of the raw continuous values as was done in the original work by Lenselink et al.[3] as well as in other binding affinity prediction benchmarks such as Davis. Another alternative to achieve a more realistic dataset, is to add more inactive interactions to the dataset such as with the strategy proposed by Mervin et al.[5]. Additionally, we note that the tails of the distribution in Fig. S1 does not appear to be symmetric, for which reason the L1 loss is more suited than the Mean Squared Error (MSE) for this particular dataset.

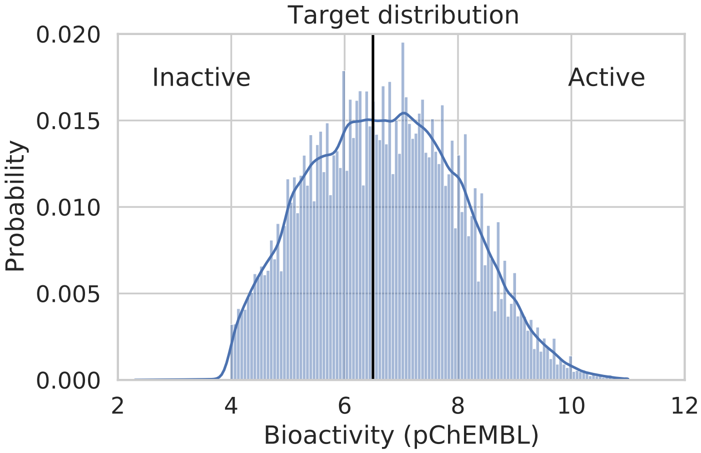

Figure S1: Distribution of bioactivity values from the full benchmark dataset derived from ChEMBL. A fixed threshold of 6.5 log affinity was imposed in the benchmark, for the purpose of creating a balanced class division into active/inactive interactions.

Complementary results for classification of bioactivity

The following supplementary material presents additional results on the Lenselink benchmark.

Full re-implemented benchmarking results

Tables S3 and S4 present the results on the Lenselink benchmark that are solely from our own experiments, whereas Table 1 in the main paper presented the top results from either our experiments or previous work. A more fair comparison can be made from the results presented in this section, as all experiments are made on the exact same data splits. However, the same conclusions as from Table 1 in the main paper can be drawn. HyperPCM outperforms all baselines significantly ($p < 0.05$, paired) in all four settings, both in terms of MCC and AUC.

Extended zero-shot analysis with unseen molecules

We hypothesize that a drawback of our method compared to previous approaches with concatenated embeddings, could be that the models for concatenated embeddings have greater capacity to memorize drug compounds seen in the training data whereas the advantage of our

Table S3: **Matthews Correlation Coefficient (MCC).** From 10-fold cross-validation in the random, LCCO, and LPO settings and 10 re-runs in the temporal setting. All results from re-implementation of baseline models. Best performance per setting is marked in bold and best baseline performance is marked in italic. Underlined results indicate statistical significance ($p < 0.05$, paired) over the best baseline on identical test sets.

	Embedding		Few-/many-shot			**Zero-shot**
Model	Drug	Target	Random	Temporal	LCCO	LPO
DeepPCM[4]	MolBert	UniRep	0.622±0.006	*0.370±0.008*	0.452±0.058	0.288±0.045
	MolBert	ProtBert	0.625±0.006	0.362±0.006	0.455±0.054	0.299±0.043
	MolBert	ProtT5	0.620±0.004	0.360±0.003	0.452±0.057	0.296±0.040
	MolBert	SeqVec	0.639±0.003	*0.370±0.006*	0.471±0.063	0.310±0.036
	CDDD	UniRep	0.623±0.006	0.352±0.009	0.451±0.064	0.298±0.039
	CDDD	ProtBert	0.635±0.004	0.343±0.006	0.462±0.060	0.294±0.049
	CDDD	ProtT5	0.634±0.005	0.353±0.006	0.460±0.056	0.310±0.054
	CDDD	SeqVec	*0.643±0.005*	0.363±0.006	*0.478±0.048*	*0.316±0.043*
HyperPCM	CDDD	SeqVec	**0.682±0.039**	**0.395±0.005**	**0.532±0.059**	**0.340±0.051**

Table S4: **Area Under the ROC-curve (AUC)**. From 10-fold cross-validation in the random, LCCO, and LPO settings and 10 re-runs in the temporal setting. All results from re-implementation of baseline models. Best performance per setting is marked in bold and best baseline performance is marked in italic. Underlined results indicate statistical significance ($p < 0.05$, paired) over the best baseline on identical test sets.

	Embedding		Few-/many-shot			**Zero-shot**
Model	Drug	Target	Random	Temporal	LCCO	LPO
DeepPCM[4]	MolBert	UniRep	0.893±0.002	0.743±0.004	0.803±0.032	0.697±0.028
	MolBert	ProtBert	0.894±0.002	0.740±0.003	0.804±0.031	0.705±0.029
	MolBert	ProtT5	0.892±0.002	0.739±0.002	0.802±0.032	0.702±0.029
	MolBert	SeqVec	*0.900±0.001*	*0.747±0.003*	0.813±0.033	0.711±0.025
	CDDD	UniRep	0.893±0.002	0.735±0.004	0.800±0.034	0.705±0.026
	CDDD	ProtBert	0.897±0.002	0.732±0.003	0.805±0.033	0.704±0.033
	CDDD	ProtT5	0.897±0.002	0.737±0.004	0.805±0.031	0.710±0.038
	CDDD	SeqVec	*0.900±0.002*	0.743±0.004	*0.814±0.027*	*0.715±0.026*
HyperPCM	CDDD	SeqVec	**0.919±0.017**	**0.765±0.003**	**0.850±0.028**	**0.738±0.030**

HyperPCM model is more prominent with regards to generalization in terms of both unseen protein targets and drug compounds. As such, we provide an extended analysis comparing the results of the best performing baseline and our HyperNetwork method on a subset of the LPO test set containing only unseen drug compounds. Fig. S2 presents the distribution of performance over the 10-fold cross-validation, on a subsets of the LPO split containing only drug compounds that were not seen during training. As expected, HyperPCM outperforms DeepPCM even more consistently on these, more demanding test sets. The performance of our method in the extended setting is 0.313 ± 0.058 compared to 0.281 ± 0.051 for the best baseline, which is a significant improvement ($p = 0.002$).

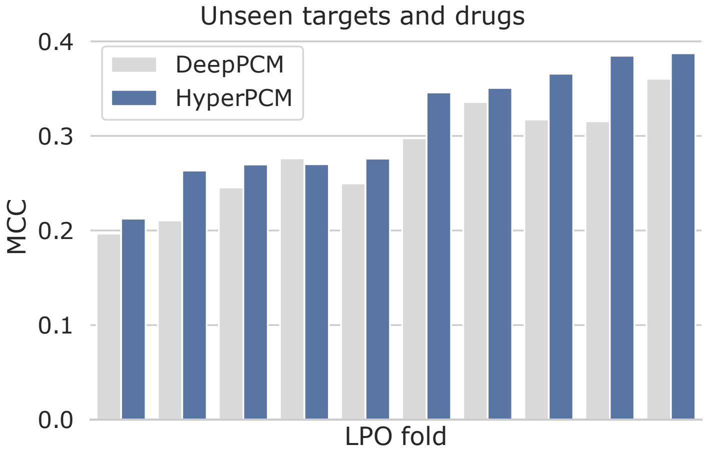

Figure S2: **Extended LPO.** Comparing test performance on each fold from the cross-validation of the baseline, DeepPCM with CDDD and SeqVec encoders, and our model, HyperPCM, on a subsets of the LPO split with only unseen drug compounds.

Additional ablation study: learning curves

As a complement to the ablation study, we present the average learning curves from the experiments of the DeepPCM baseline and our HyperPCM model using the L1 loss compared to the BCE loss in Fig. S3. The results suggest that both models overfit immediately in the binary classification case, whereas generalization continues to improve for at least 200 epochs using L1. Note that the trained models using during inference are always chosen according

to an early stopping criteria in terms of optimal validation performance.

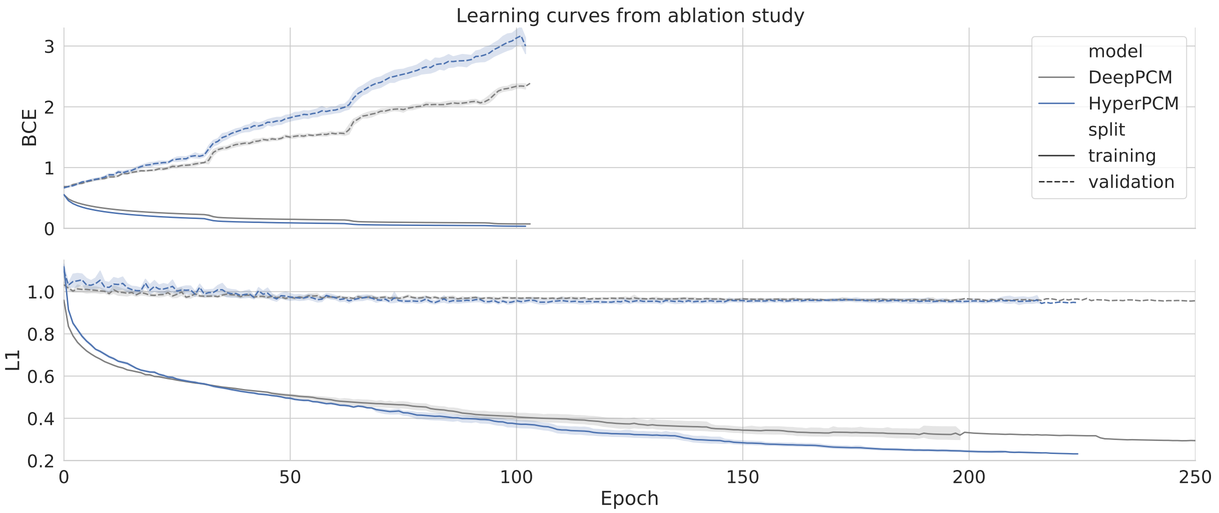

Figure S3: **Learning curves.** Learning curves from ablation study comparing the DeepPCM baseline and HyperPCM, trained using Binary Cross-Entropy (BCE) loss versus L1 loss.

Complementary results for drug–target binding affinity prediction

In the following supplementary material additional results in terms of MSE on the Davis benchmark is presented in Table S5 for the splitting strategies proposed by Nguyen et al.[6]. Note that the results references from Nguyen et al.[6] have been transferred from RMSE to MSE. Additionally, Table S6 gives a more extensive overview of all models evaluated using the 5-fold cross-validation in the random split proposed by Öztürk et al.[7]. These results differ from the ones on Table 3 and S5 due to slight differences in how the datasets where created and due to the multiple folds provided by Öztürk et al.[7].

Table S5: **Davis benchmark.** Average MSE from five re-runs with varying random seeds. Standard deviation displayed when available. The best performance per setting is marked in bold. Underlined results indicate statistical significance ($p < 0.05$, paired) over the other models.

	Embedding		Few-/many-shot		**Zero-shot**	
Model	Drug	Target	Random	Cold-drug	Cold-target	Cold
GraphDTA[8]	GCN	CNN	0.284	0.945	0.500	1.130
GraphDTA[8]	GIN	CNN	0.257	0.920	0.510	1.134
DGraphDTA[9]	GCN	GCN	0.242	0.918	0.470	1.155
GLFA[6]	GCN	TAPE	0.235	0.861	0.453	1.144
GEFA[6]	GCN	TAPE	**0.228**	0.847	0.433	0.990
RF	CDDD	SeqVec	0.323±0.002	0.775±0.006	<u>**0.405±0.004**</u>	**0.909±0.011**
XGBoost	CDDD	SeqVec	0.261±0.001	**0.768±0.023**	0.419±0.002	0.916±0.012
HyperPCM	CDDD	SeqVec	0.234±0.004	0.769±0.025	0.432±0.008	0.915±0.024

Results for the GraphDTA, DGraphDTA, GLFA, and GEFA models are repeated from Nguyen et al.[6].

Table S6: **Davis benchmark.** Extensive overview of performances for models evaluated on the pre-defined 5-fold cross-validation proposed by Öztürk et al.[7] in the random setting of the Davis benchmark. Standard deviation is displayed when available. The best performance per metric is marked in bold.

	Embedding		Random split			
Model	Drug	Target	MSE ↓	CI ↑	ROC-AUC ↑	PR-AUC ↑
RF[10]	ECFP	PSC	0.359±0.003	0.854±0.002		
SVM[10]	ECFP	PSC	0.383±0.002	0.857±0.001		
DNN[10,11]	ECFP	PSC	0.244±0.009	0.893±0.003	0.864±0.009	0.258±0.024
LR[11]	ECFP	PSC			0.835±0.010	0.232±0.023
DeepConvDTI[12]	ECFP	CNN			0.884±0.008	0.299±0.039
DeepDTA[7]	CNN	CNN	0.261	0.878±0.004	0.880±0.007	0.302±0.044
DeepCPI (GNN-CPI)[13]	GNN	CNN	0.293	0.867	0.840±0.012	0.705
GraphDTA[8]	GCN	CNN	0.254	0.880	0.874	0.289
GraphDTA[8]	GIN	CNN	0.229	0.893		
DGraphDTA[9]	GCN	GCN	**0.202**	0.904		
GLFA[6]	GCN	TAPE	0.235	0.895		
GEFA[6]	GCN	TAPE	0.228	0.893		
MGraphDTA[10]	GCN	CNN	0.207±0.001	0.900±0.004		
TransformerCPI[14]	GCN	CNN			0.874	0.277
MolTrans[11]	FCS	FCS			0.907±0.002	0.404±0.016
DeepFusion[15]	Transformer*				0.911	0.402
Affinity2Vec[2]	Seq2seq	ProtVec	0.240	0.887		0.734
FusionDTA[16]	BiLSTM	ESM-1b	0.208	**0.913±0.002**		
RF	CDDD	SeqVec	0.332±0.005	0.863±0.002	0.929±0.002	0.653±0.006
XGBoost	CDDD	SeqVec	0.267±0.004	0.888±0.002	0.952±0.001	0.713±0.006
HyperPCM	CDDD	SeqVec	0.231±0.008	0.894±0.002	**0.958±0.002**	**0.740±0.015**

FCS: Frequent Consecutive Sub-sequence Mining PSC: Protein Sequence Composition
LR: Logistic Regression * DeepFusion uses a mix of local features extracted using a Transformer and global features extracted with similarity-based approaches, similarly for both drugs and targets.

References

(1) Pedregosa, F.; Varoquaux, G.; Gramfort, A.; Michel, V.; Thirion, B.; Grisel, O.; Blondel, M.; Prettenhofer, P.; Weiss, R.; Dubourg, V.; Vanderplas, J.; Passos, A.; Cournapeau, D.; Brucher, M.; Perrot, M.; Duchesnay, E. Scikit-learn: Machine Learning in Python. *J. Mach. Learn. Res.* **2011**, *12*, 2825–2830.

(2) Thafar, M. A.; Alshahrani, M.; Albaradei, S.; Gojobori, T.; Essack, M.; Gao, X. Affinity2Vec: Drug-Target Binding Affinity Prediction Through Representation Learning, Graph Mining, and Machine Learning. *Sci. Rep.* **2022**, *12*, 1–18.

(3) Lenselink, E. B.; Ten Dijke, N.; Bongers, B.; Papadatos, G.; Van Vlijmen, H. W.; Kowalczyk, W.; IJzerman, A. P.; Van Westen, G. J. Beyond the Hype: Deep Neural Networks Outperform Established Methods Using a ChEMBL Bioactivity Benchmark Set. *J. Cheminf.* **2017**, *9*, 1–14.

(4) Kim, P. T.; Winter, R.; Clevert, D.-A. Unsupervised Representation Learning for Proteochemometric Modeling. *Int. J. Mol. Sci.* **2021**, *22*, 12882.

(5) Mervin, L. H.; Afzal, A. M.; Brive, L.; Engkvist, O.; Bender, A. Extending In Silico Protein Target Prediction Models to Include Functional Effects. *Front. Pharmacol.* **2018**, *9*, 613.

(6) Nguyen, T. M.; Nguyen, T.; Le, T. M.; Tran, T. GEFA: Early Fusion Approach in Drug-Target Affinity Prediction. *IEEE/ACM Trans. Comput. Biol. Bioinf.* **2021**, *19*, 718–728.

(7) Öztürk, H.; Özgür, A.; Ozkirimli, E. DeepDTA: Deep Drug–Target Binding Affinity Prediction. *Bioinform.* **2018**, *34*, i821–i829.

(8) Nguyen, T.; Le, H.; Quinn, T. P.; Nguyen, T.; Le, T. D.; Venkatesh, S. GraphDTA:

Predicting Drug–Target Binding Affinity with Graph Neural Networks. *Bioinform.* **2021**, *37*, 1140–1147.

(9) Jiang, M.; Li, Z.; Zhang, S.; Wang, S.; Wang, X.; Yuan, Q.; Wei, Z. Drug–Target Affinity Prediction Using Graph Neural Network and Contact Maps. *RSC Adv.* **2020**, *10*, 20701–20712.

(10) Yang, Z.; Zhong, W.; Zhao, L.; Chen, C. Y.-C. MGraphDTA: Deep Multiscale Graph Neural Network for Explainable Drug–Target Binding Affinity Prediction. *Chem. Sci.* **2022**, *13*, 816–833.

(11) Huang, K.; Xiao, C.; Glass, L. M.; Sun, J. MolTrans: Molecular Interaction Transformer for Drug–Target Interaction Prediction. *Bioinform.* **2021**, *37*, 830–836.

(12) Lee, I.; Keum, J.; Nam, H. DeepConv-DTI: Prediction of Drug–Target Interactions via Deep Learning with Convolution on Protein Sequences. *PLoS Comput. Biol.* **2019**, *15*, e1007129.

(13) Tsubaki, M.; Tomii, K.; Sese, J. Compound–Protein Interaction Prediction with End-To-End Learning of Neural Networks for Graphs and Sequences. *Bioinform.* **2019**, *35*, 309–318.

(14) Chen, L.; Tan, X.; Wang, D.; Zhong, F.; Liu, X.; Yang, T.; Luo, X.; Chen, K.; Jiang, H.; Zheng, M. TransformerCPI: Improving Compound–Protein Interaction Prediction by Sequence-Based Deep Learning with Self-Attention Mechanism and Label Reversal Experiments. *Bioinform.* **2020**, *36*, 4406–4414.

(15) Song, T.; Zhang, X.; Ding, M.; Rodriguez-Paton, A.; Wang, S.; Wang, G. DeepFusion: A Deep Learning Based Multi-Scale Feature Fusion Method for Predicting Drug-Target Interactions. *Methods* **2022**, *204*, 269–277.

(16) Yuan, W.; Chen, G.; Chen, C. Y.-C. FusionDTA: Attention-Based Feature Polymerizer and Knowledge Distillation for Drug-Target Binding Affinity Prediction. *Briefings Bioinf.* **2022**, *23*, bbab506.
